# Effect of ancillary drugs on sevoflurane related emergence agitation in children undergoing ophthalmic surgery: a Bayesian network meta-analysis

**DOI:** 10.1186/s12871-019-0810-y

**Published:** 2019-08-01

**Authors:** Dan Tan, Haifa Xia, Shujun Sun, Fuquan Wang

**Affiliations:** 1 Editorial Office, Journal of New Medicine, Zhongnan Hospital of Wuhan University, No.169 Donghu Road, Wuchang District, Wuhan, 430071 Hubei Province China; 2 Department of Anesthesiology, Institute of Anesthesia and Critical Care, Union Hospital, Tongji Medical College, Huazhong University of Science and Technology, Wuhan, China

**Keywords:** Ophthalmic surgery, Emergence agitation, Anesthetic drugs, Network meta-analysis, Randomized control trial

## Abstract

**Background:**

The comparative efficacy of ancillary drugs on sevoflurane related emergence agitation (EA) in children undergoing ophthalmic surgery remains controversial.

**Methods:**

The databases were retrieved in an orderly manner from the dates of their establishment to October, 2018, including PubMed, The Cochrane Library and Web of Science, to collect randomized controlled trials (RCT) of different anesthetic drugs combined with sevoflurane for ophthalmic surgery. Then a network meta-analysis was conducted using R and Stata 12.0 softwares.

**Results:**

The meta-analysis showed that, in reducing sevoflurane related EA, dexmedetomidine, ketamine, propofol, fentanyl, midazolam, sufentanil, remifentanil and clonidine were superior to placebo (*P* < 0.05). The network meta-analysis showed that the effects of ancillary drugs combine with sevoflurane in reducing risk of EA in children undergoing ophthalmic surgery was superior to placebo: dexmedetomidine (OR = 0.17, 95% CrI 0.12–0.22), ketamine (OR = 0.30, 95% CrI 0.11–0.49), propofol (OR = 0.24, 95% CrI 0.09–0.63), fentanyl (OR = 0.16, 95% CrI 0.08–0.56), midazolam (OR = 0.20, 95% CrI 0.09–0.40), sufentanil (OR = 0.27, 95% CrI 0.14–0.41), remifentanil (OR = 0.18, 95% CrI 0.08–0.54) and clonidine (OR = 0.14, 95% CrI 0.07–0.41). The SUCRA of placebo, dexmedetomidine, ketamine, propofol, fentanyl, midazolam, sufentanil, remifentanil, clonidine were respectively 0.26, 77.93, 27.71, 42.8, 69.43, 52.89, 59.83, 57.62 and 61.53%.

**Conclusions:**

The effects of dexmedetomidine combine with sevoflurane in reducing risk of emergence agitation in children undergoing ophthalmic surgery was superior to other drugs.

## Background

Ophthalmic surgery is one of the common operations in children [1]. In ophthalmic surgery, especially in children’s ophthalmology, the operation time is short, and the patient’s self-control ability is weak, so the quality of anesthesia is required to be high [2]. It is a necessary condition for the operation to effectively inhibit the stress response and oculocardiac reflex caused by the operation under the anesthesia. Moreover, children may experience severe adverse events during the course of anesthesia, for instance, cardiac arrest, bronchial hyperreactivity, upper respiratory tract infection, and obstructive sleep apnea. Therefore, the selection of appropriate anesthetic inducing drugs is of great significance for the implementation of the operation [3, 4].

Sevoflurane is the most commonly used inhalation anesthetic in pediatric anesthesia [5]. It has the characteristics of fast induction, rapid clearance, rapid awakening and easy adjustment of anesthesia depth. In addition, the drug has little effect on heart rate, airway stimulation is also very small, can achieve the role of relaxation of smooth muscle [6]. However, when it is used as the only anesthetic, it is associated with a high incidence of emergence agitation (EA) and may be harmful to patients [7–9]. Anesthetic adjuvants such as metomidine, ketamine, propofol, fentanyl, midazolam, sufentanil, remifentanil, clonidine and other drugs have been effectively used to prevent EA. However, these drugs may increase the sedative effect after anesthesia, leading to slow awakening and, in some cases, adverse side effects, such as nausea and vomiting [10, 11]. Studies have shown that the combination of anesthetic adjuvant and sevoflurane can produce synergistic effect, not only maintain good anesthetic effect, rapid recovery after operation, but also do not cause respiratory inhibition. It can maintain the analgesic effect for a long time after operation, and effectively reduce the EA, crying and other adverse reactions in children [12].

In this study, we tried to investigate eight adjuvant drugs in combination with sevoflurane in children undergoing ophthalmic surgery. We use a Bayesian network to determine which adjuvant drugs combine with sevoflurane can affect the incidence of EA in children undergoing ophthalmic surgery.

## Methods and analysis

### Eligibility criteria

Eligibility criteria will be designed according to the PICOS (Participant-Intervention-Comparator-Outcome-Study design) framework.

### Selection of studies

We will include studies assessing the effect of different anesthetic drugs combined with sevoflurane for ophthalmic surgery.

### Study design

We only include randomized controlled trials.

### Participants

We will include patients with undergoing ophthalmic surgery and those receiving sevoflurane under 0 and 18 years.

### Interventions

The control group was given an anesthetic adjuvant or placebo, and the experimental group was given an anesthetic adjuvant.

### Outcome measurements

Contains the main outcome indicator is number of patients with EA. EA is known as emergence delirium which is often accompanied with revival after pediatric anesthesia.

### Search strategy

By using the combination of subject words and free words, the databases were retrieved in an orderly manner from the dates of their establishment to October, 2018, including PubMed, The Cochrane Library and Web of Science, with keywords including “Ophthalmic surgery” [MeSH] OR “Eye surgery” [MeSH] AND “Anesthetic Drugs” [MeSH] OR “Anesthetic Agents” [MeSH] OR “Anesthetic Effect” [MeSH] OR “Dexmedetomidine” [MeSH] OR “Ketamine” [MeSH] OR “Propofol” [MeSH] OR “Fentanyl” [MeSH] OR “Midazolam” [MeSH] OR “Sufentanil” [MeSH] OR “Remifentanil” [MeSH] OR “Clonidine” [MeSH] AND “sevoflurane” [MeSH] AND “Randomized Controlled Trial” [MeSH] OR “RCT” [MeSH].

### Data extraction

According to the inclusion criteria, the titles and abstracts of the literature were screened by two researchers independently of each other, and the unrelated literature was eliminated. Then through reading the full text, exclude the literature that does not accord with this research scheme, and record the reasons and quantity of exclusion. Finally, the selected literature was cross-checked by two researchers. Using Excel 2013 Software design data extraction table to extract the key information in the literature after the inclusion of the literature.

### Risk of bias assessment

The quality of included literature was evaluated by Cochrane collaboration network evaluation risk tool. The quality of literature was evaluated according to random method, distribution concealment, blind method, incomplete outcome data, selective outcome report and other biased sources.

### Statistical analysis

Stata 12.0 software was used for statistical analysis. χ^2^ test was used to analyze the heterogeneity among the studies, and I^2^ was used for quantitative analysis. If I^2^ < 50%, it indicated that there was homogeneity among the studies, which could be directly combined and analyzed by fixed effect model. If I^2^ ≥ 50%, the heterogeneity of each study is indicated, and the random effect model is used for statistical analysis [13]. The biggest difference between the network meta analysis and the traditional Meta analysis is that it can compare multiple intervention measures at the same time. The two interventions which do not have direct comparison are indirectly compared and quantitatively analyzed through the mesh relationship, and the best scheme is obtained according to the advantages and disadvantages of the outcome index. Bayesian network model based on Markov chain Monte Carlo operation for analyzing the therapeutic effects of drugs in two groups and multiple groups. All the included drugs were sorted using the surface under the cumulative ranking (SUCRA) to determine the pros and cons of the drug treatment on sevoflurane related EA in children undergoing ophthalmic surgery. The larger the SUCRA, the better the effect. Bayesian network analysis using R software.

## Results

### Literature search results

**Fig. 1:**
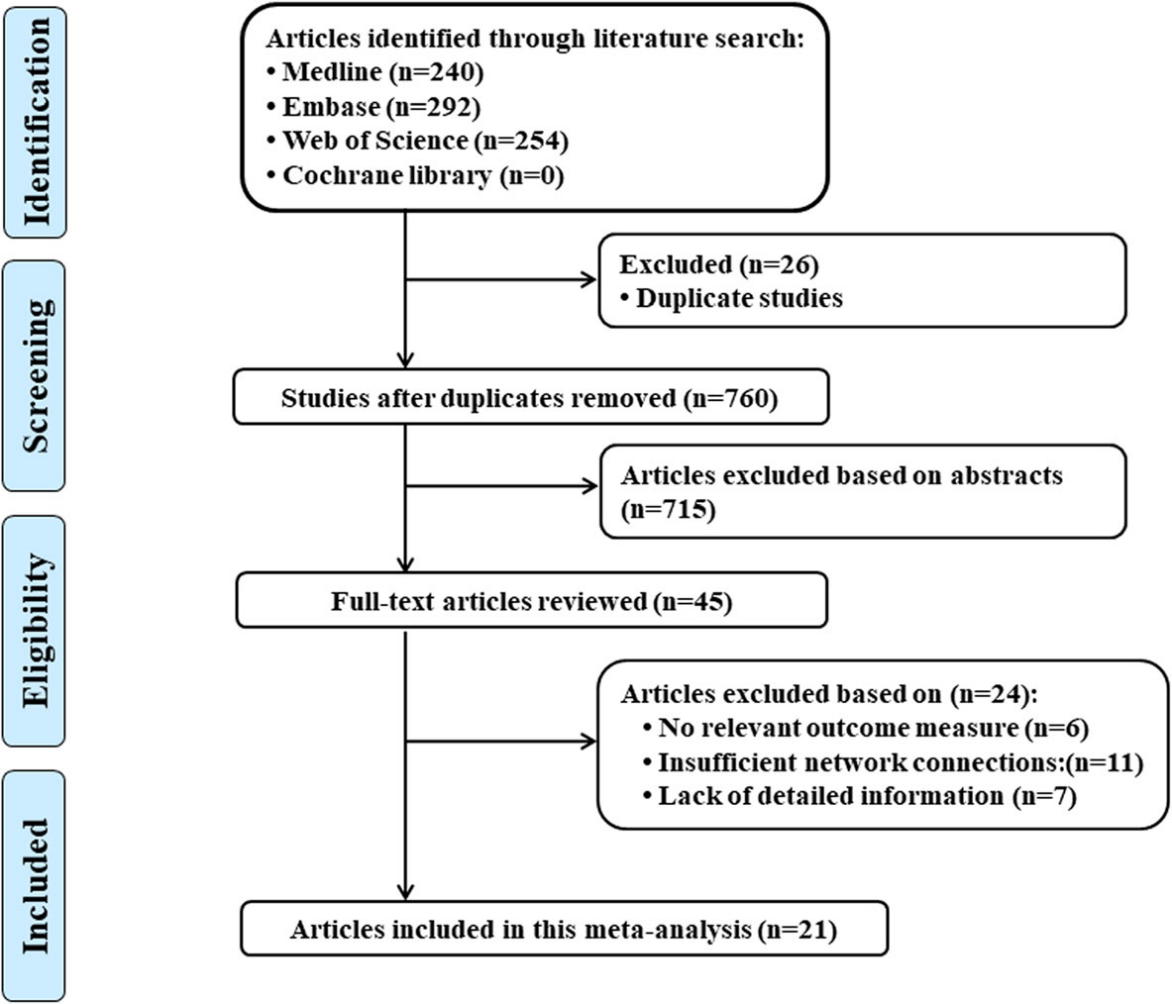
Flow diagram of the study selection process

**Fig. 2:**
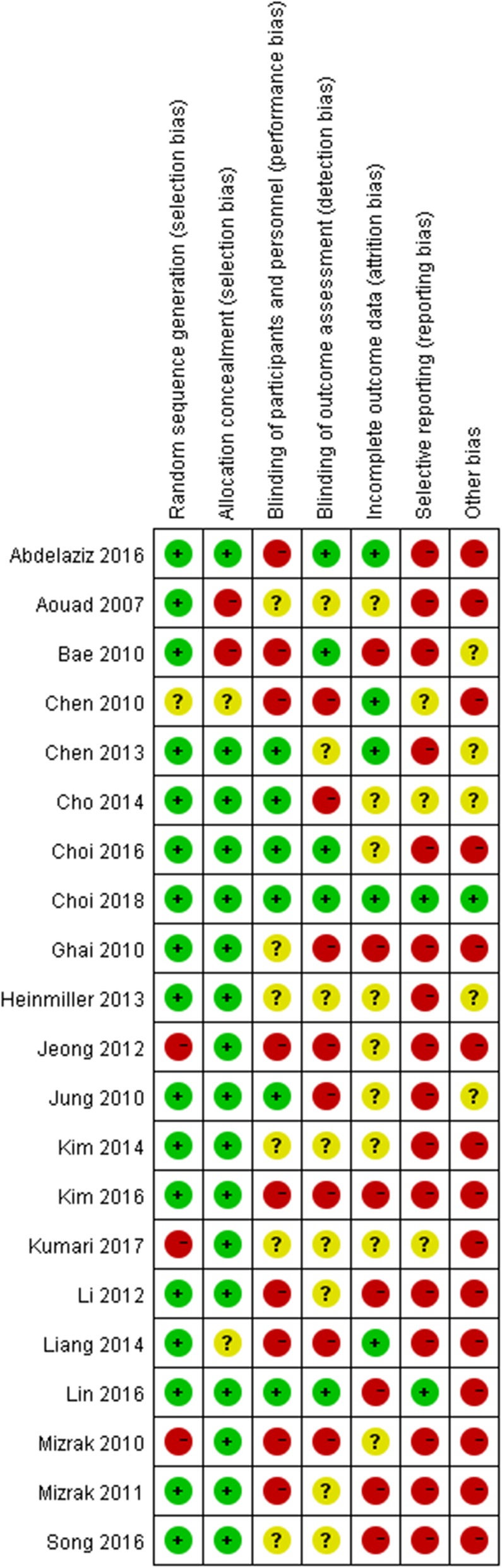
Risk of bias of the included RCTs (Review authors’ judgments about each risk of bias item for each included study. +, low risk; −, high risk;?, unclear risk)

**Fig. 3:**
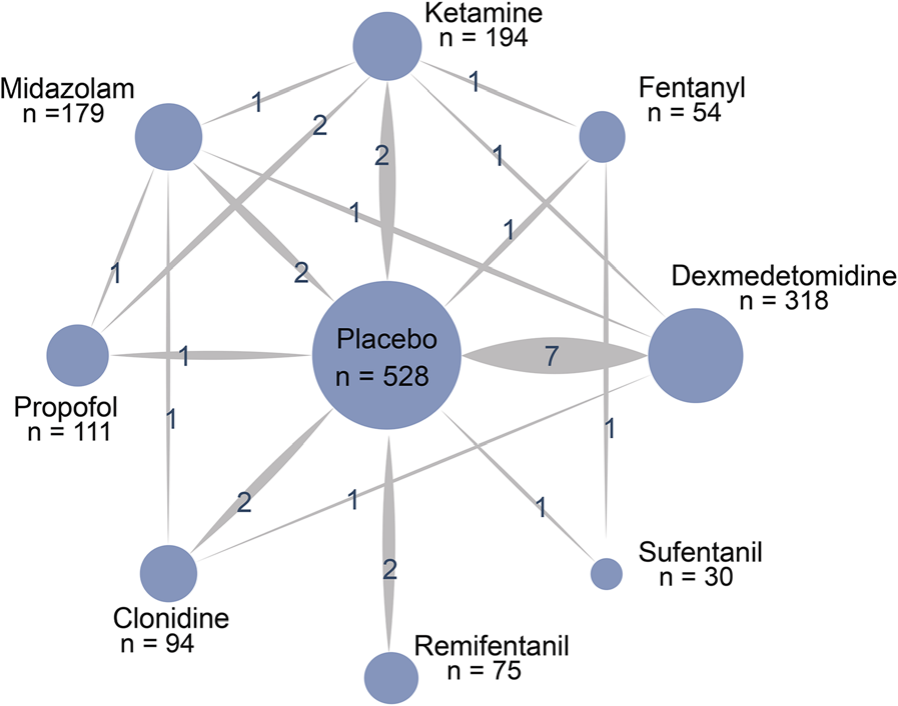
Network of randomized controlled trials comparing different adjuvant therapies for EA in ophthalmic surgery. The thickness of the connecting lines represents the number of trials between each comparator, and the size of each node corresponds to the number of subjects who received the same pharmacological agent (sample size)

A total of 240 studies from Medline, 292 studies from Embase and 254 studies from Web of Science. After removing duplicates study, 760 studies were identifed. After reviewing their titles and abstracts, 715 citations were excluded. The remaining 45 citations were assessed in more detail for eligibility by reading the full text. Among them, 6 studies were excluded due to no relevant outcome measure; 11 studies were excluded due to insufficient network connections; 7 study was excluded due to lack of detailed information. Finally, 21 studies were used for the final data synthesis [14–34]. The flow chart of literature searching was presented in Fig. 1. Figure 2 showed the risk of bias of 21 studies included in this meta-analysis. The characteristics of the included studies are shown in the Table 1. Figure 3 showed the pattern of evidence within the network is displayed.

**Table 1:** Characteristics of included studies

Author	Year	Treatments											
		Treatments 1	Age (year)	Male (%)	Cases /n	Treatments 2	Age (year)	Male (%)	Cases /n	Treatments 3	Age (year)	Male (%)	Cases /n
Aouad et al	2007	Propofol	4.2 ± 1.4	46	8/41	Placebo	4.3 ± 1.3	58	17/36				
Bae et al	2010	Midazolam	4.9 ± 1.6	53	2/15	Placebo	4.1 ± 1.4	47	19/45				
Chen et al	2010	Midazolam	3.6 ± 1.9	–	5/40	Propofol	3.7 ± 1.7	–	8/40	Ketamine	3.8 ± 2.0	–	15/40
Chen et al	2013	Dexmedetomidine	4.1 ± 1.3	63	3/28	Ketamine	4.2 ± 1.2	67	6/28	Placebo	4.3 ± 1.1	62	11/28
Cho et al	2014	Midazolam	8.0 ± 2.1	33	10/60	Placebo	8.0 ± 2.1	30	13/30				
Choi et al	2018	Remifentanil	6.0 ± 1.1	44	2/39	Placebo	5.6 ± 1.1	49	14/41				
Choi et al	2016	Placebo	6.1 ± 2.3	48	21/33	Remifentanil	6.2 ± 2.0	50	11/34				
Jeong et al	2012	Placebo	4.8 ± 0.4	55	15/20	Ketamine	5.0 ± 0.4	50	10/40				
Jung et al	2010	Ketamine	5.4 ± 1.9	48	4/23	Fentanyl	7.5 ± 2.0	33	0/24				
Kim et al	2014	Dexmedetomidine	4.3 ± 1.4	38	7/47	Placebo	4.3 ± 1.0	55	33/47				
Kim et al	2016	Midazolam	4.1 ± 1.4	47	15/34	Ketamine	4.2 ± 1.3	48	11/33				
Liang et al	2014	Sufentanil	5.1 ± 1.3	57	9/30	Fentanyl	4.8 ± 1.3	47	11/30	Placebo	5.5 ± 1.4	43	19/30
Li et al	2012	Dexmedetomidine	5.0 ± 2.0	53	3/30	Placebo	4.0 ± 1.0	57	13/30				
Lin et al	2016	Dexmedetomidine	4.7 ± 1.9	60	10/60	Placebo	4.1 ± 1.6	50	24/30				
Mizrak et al	2010	Ketamine	7.7 ± 3.1	37	5/30	Propofol	6.9 ± 3.0	40	5/30				
Mizrak et al	2011	Dexmedetomidine	8.5 ± 2.6	50	6/30	Placebo	8.6 ± 2.8	43	16/30				
Song et al	2016	Dexmedetomidine	4.3 ± 1.7	50	6/28	Placebo	3.8 ± 1.5	50	17/28				
Abdelaziz et al	2016	Dexmedetomidine	2.7 ± 1.5	52	4/35	Midazolam	2.5 ± 1.2	52	7/35	Placebo	2.8 ± 1.7	56	15/35
Kumari et al	2017	Dexmedetomidine	7.9 ± 3.2	57	3/30	Clonidine	7.5 ± 2.9	63	1/30	Midazolam	6.6 ± 2.8	60	0/30
Heinmiller et al	2013	Clonidine	4.3 ± 1.5	40	6/25	Placebo	4.1 ± 1.3	48	15/25				
Ghai et al	2010	Clonidine	3.4 ± 1.5	62	3/39	Placebo	3.0 ± 1.4	60	16/40

### Results of pairwise meta-analysis

Table 2 displayed the results produced by pairwise meta-analysis. The effects of ancillary drugs combine with sevoflurane in reducing risk of EA in children undergoing ophthalmic surgery was superior to placebo: dexmedetomidine(OR = 0.26, 95% CrI 0.17–0.39), ketamine (OR = 0.41, 95% CrI 0.20–0.86), propofol (OR = 0.39, 95% CrI 0.18–0.83), fentanyl (OR = 0.56, 95% CrI 0.29–0.89), midazolam (OR = 0.40, 95% CrI 0.21–0.75), sufentanil (OR = 0.47, 95% CrI 0.38–0.58), remifentanil (OR = 0.35, 95% CrI 0.17–0.73) and clonidine (OR = 0.29, 95% CrI 0.13–0.66).

**Table 2:** Summary odds ratios of emergence agitation and heterogeneity for each direct comparison

Comparison	OR (95% CI)	P-heterogeneity	I-squared	Tau-squared
Dexmedetomidine vs. Placebo	*0.26 (0.17, 0.39)*	0.697	0.0%	< 0.001
Ketamine vs. Placebo	*0.41 (0.20, 0.86)*	0.514	0.0%	0.018
Propofol vs. Placebo	*0.39 (0.18, 0.83)*	–	–	< 0.001
Fentanyl vs. Placebo	*0.56 (0.29, 0.89)*	–	–	< 0.001
Midazolam vs. Placebo	*0.40 (0.21, 0.75)*	0.912	0.0%	0.004
Sufentanil vs. Placebo	*0.47 (0.38, 0.58)*	–	–	< 0.001
Remifentanil vs. Placebo	*0.35 (0.17, 0.73)*	0.173	46.1%	0.005
Clonidine vs. Placebo	*0.29 (0.13, 0.66)*	0.399	0.0%	0.003
Ketamine vs. Dexmedetomidine	2.00 (0.46, 8.80)	–	–	0.359
Midazolam vs. Dexmedetomidine	0.97 (0.33, 2.83)	0.126	47.3%	0.851
Clonidine vs. Dexmedetomidine	0.33 (0.03, 3.39)	–	–	0.353
Propofol vs. Ketamine	0.66 (0.30, 1.43)	0.455	0.0%	0.292
Fentanyl vs. Ketamine	0.11 (0.05, 2.09)	–	–	0.140
Midazolam vs. Ketamine	0.73 (0.37, 1.43)	0.159	46.3%	0.360
Midazolam vs. Propofol	0.63 (0.19, 2.07)	–	–	0.443
Sufentanil vs. Fentanyl	0.82 (0.30, 2.26)	–	–	0.699
Clonidine vs. Midazolam	0.96 (0.17, 5.60)	–	–	0.506

*P* value less than 0.05 is considered as significance with italic fonts

### Network meta-analysis

**Fig. 4:**
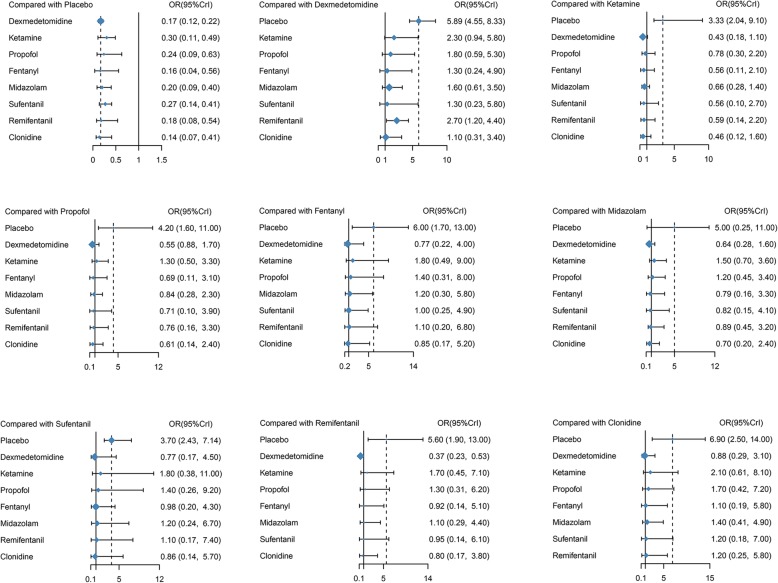
Forest plots of odds ratios (95% creditable intervals) produced by network meta-analysis

Table 3 displayed the results produced by network meta-analysis. The effects of ancillary drugs combine with sevoflurane in reducing risk of EA in children undergoing ophthalmic surgery was superior to placebo: dexmedetomidine(OR = 0.17, 95% CrI 0.12–0.22), ketamine (OR = 0.30, 95% CrI 0.11–0.49), propofol (OR = 0.24, 95% CrI 0.09–0.63), fentanyl (OR = 0.16, 95% CrI 0.08–0.56), midazolam (OR = 0.20, 95% CrI 0.09–0.40), sufentanil (OR = 0.27, 95% CrI 0.14–0.41), remifentanil (OR = 0.18, 95% CrI 0.08–0.54) and clonidine (OR = 0.14, 95% CrI 0.07–0.41)(Fig. 4).

**Table 3:** Network meta-analysis comparisons

	Placebo	Dexmedetomidine	Ketamine	Propofol	Fentanyl	Midazolam	Sufentanil	Remifentanil	Clonidine
Placebo	1	*5.89 (4.55, 8.33)*	*3.33 (2.04, 9.10)*	*4.20 (1.60, 11.0)*	*6.00 (1.70, 13.0)*	*5.00 (2.50, 11.0)*	*3.70 (2.43, 7.14)*	*5.60 (1.90, 13.0)*	*6.90 (2.50, 14.0)*
Dexmedetomidine	*0.17 (0.12, 0.22)*	1	0.43 (0.18, 1.10)	0.55 (0.18, 1.70)	0.77 (0.20, 4.00)	0.64 (0.28, 1.60)	0.77 (0.17, 4.50)	*0.37 (0.23, 0.53)*	0.88 (0.29, 3.10)
Ketamine	*0.30 (0.11, 0.49)*	2.30 (0.94, 5.80)	1	1.30 (0.50, 3.30)	1.80 (0.49, 9.00)	1.50 (0.70, 3.60)	1.80 (0.38, 11.0)	1.70 (0.45, 7.10)	2.10 (0.61, 8.10)
Propofol	*0.24 (0.09, 0.63)*	1.80 (0.59, 5.30)	0.78 (0.30, 2.20)	1	1.40 (0.31, 8.00)	1.20 (0.45, 3.40)	1.40 (0.26, 9.20)	1.30 (0.31, 6.20)	1.70 (0.42, 7.20)
Fentanyl	*0.16 (0.08, 0.56)*	1.30 (0.24, 4.90)	0.56 (0.11, 2.10)	0.69 (0.11, 3.10)	1	0.79 (0.16, 3.30)	0.98 (0.20, 4.30)	0.92 (0.14, 5.10)	1.10 (0.19, 5.80)
Midazolam	*0.20 (0.09, 0.40)*	1.60 (0.61, 3.50)	0.66 (0.28, 1.40)	0.84 (0.28, 2.30)	1.20 (0.30, 5.80)	1	1.20 (0.24, 6.70)	1.10 (0.29, 4.40)	1.40 (0.41, 4.90)
Sufentanil	*0.27 (0.14, 0.41)*	1.30 (0.23, 5.80)	0.56 (0.10, 2.70)	0.71 (0.10, 3.90)	1.00 (0.25, 4.90)	0.82 (0.15, 4.10)	1	0.95 (0.14, 6.10)	1.20 (0.18, 7.00)
Remifentanil	*0.18 (0.08, 0.54)*	*2.70 (1.20, 4.40)*	0.59 (0.14, 2.20)	0.76 (0.16, 3.30)	1.10 (0.20, 6.80)	0.89 (0.22, 3.40)	1.10 (0.17, 7.40)	1	1.20 (0.25, 5.80)
Clonidine	*0.14 (0.07, 0.41)*	1.10 (0.31, 3.40)	0.46 (0.12, 1.60)	0.61 (0.14, 2.40)	0.85 (0.17, 5.20)	0.70 (0.20, 2.40)	0.86 (0.14, 5.70)	0.80 (0.17, 3.80)	1

*P* value less than 0.05 is considered as significance with italic fonts

**Fig. 5:**
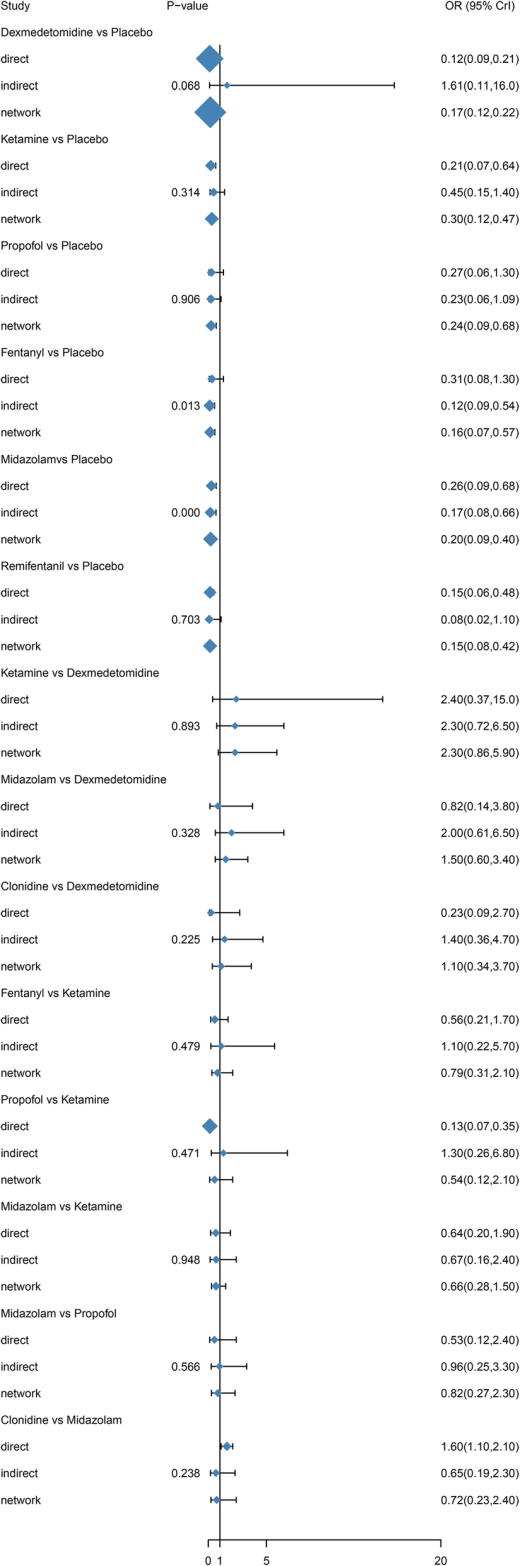
Surface under the Cumulative Ranking Curve (SUCRA), expressed as percentages, ranking the therapeutic effects and safety of treatments for EA in ophthalmic surgery. For efcacy and safety assessment, the pharmacological agent with the highest SUCRA value would be the most efcacious and safe treatment

The corresponding results of SUCRA values are presented in Fig. 5. The SUCRA of placebo, dexmedetomidine, ketamine, propofol, fentanyl, midazolam, sufentanil, remifentanil, clonidine were respectively 0.26, 77.93, 27.71, 42.8, 69.43, 52.89, 59.83, 57.62 and 61.53%. The effects of dexmedetomidine combine with sevoflurane in reducing risk of emergence agitation in children undergoing ophthalmic surgery was superior to other drugs.

### Consistency, publication bias of included studies

**Fig. 6:**
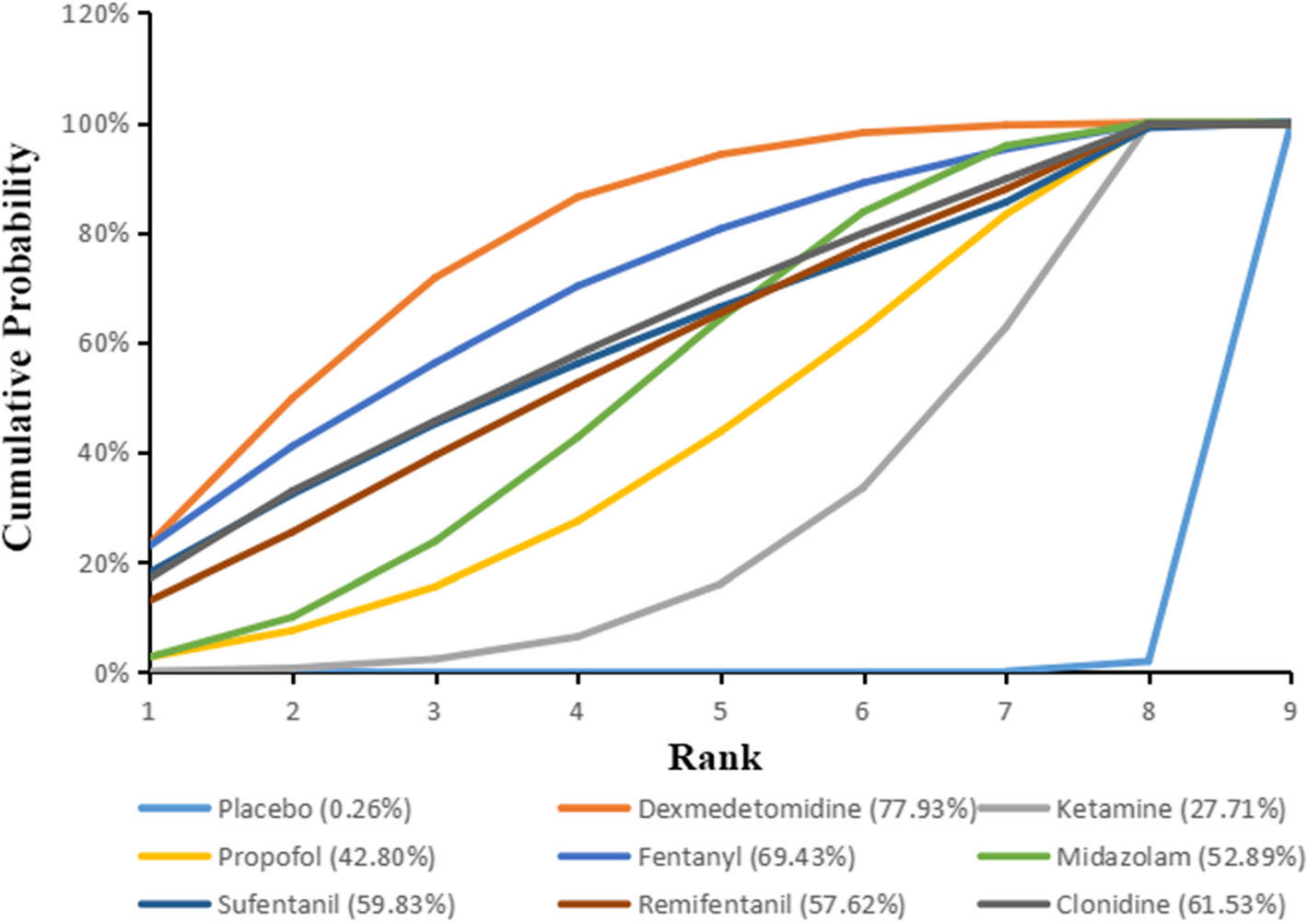
Node splitting results for each comparison

**Fig. 7:**
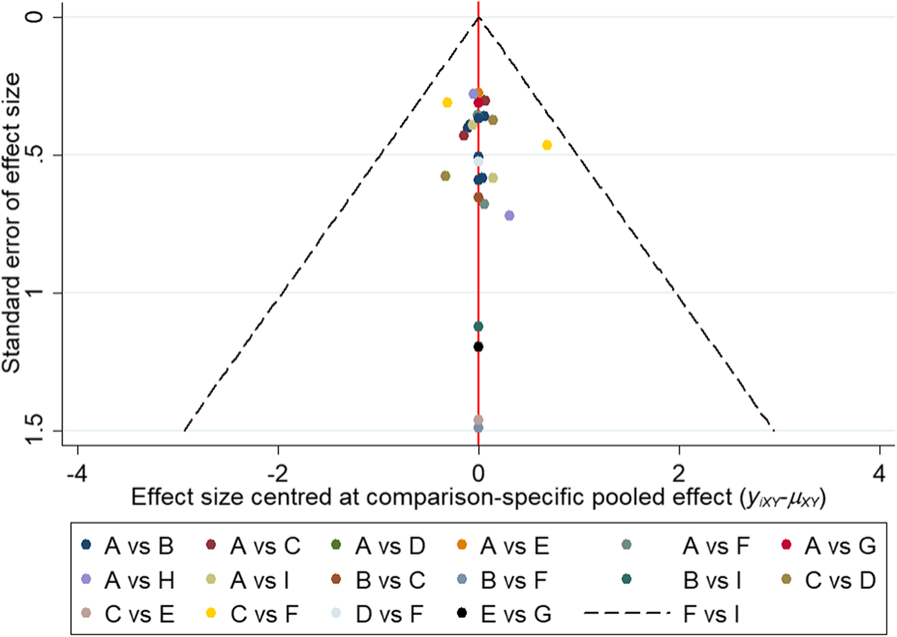
Comparison-adjusted funnel plot for the network meta-analysis. The red line suggests the null hypothesis that the study-specifc effect sizes do not differ from the respective comparison-specifc pooled effect estimates. Different colors represent different comparisons

One of the main assumptions of the network meta-analysis is the consistency between direct evidence and indirect evidence. The degree of indirect evidence is consistent with direct evidence by the node splitting method. The evidence in the network seems to be consistent with most comparisons (*P* > 0.05)(Fig. 6). All data points are evenly distributed on both sides of the inverted funnel plot, suggesting that there is less likelihood of publication bias (Fig. 7).

## Discussion

The anesthetics used in pediatric ophthalmic surgery can meet the requirements of fast effect, stable effect, quick awakening, easy adjustment, small stimulation and low incidence of complications [35, 36]. Although sevoflurane is the most commonly used inhaled anesthetic in pediatric anesthesia and it has incomparable advantages over other anesthetic in the induction period of pediatric surgical anesthesia, sevoflurane alone can lead to high risk of EA. EA during awakening refers to a state of mind in which consciousness and behavior are separated from the awakening of general anesthesia, manifested as inability to appease and irritability. The incidence of EA in children undergoing ophthalmic surgery under anesthesia is high [37]. In clinical practice, other anesthetic adjuvants can be combined with sevoflurane to prevent EA. However, the results are contradictory.

This network meta-analysis attempted to explain the effectiveness of ancillary drugs employed in sevoflurane related emergence agitation in children undergoing ophthalmic surgery treatment. Our analysis suggests that the effects of dexmedetomidine on sevoflurane related emergence agitation in children undergoing ophthalmic surgery were superior to other drugs, and fentanyl was close behind. Dexmetomidine is a novel, highly selective α2 adrenergic receptor agonist, which acts on α2 adrenergic receptor and inhibits sympathetic nerve activity [38]. It can produce sedative and analgesic effects, reduce the dose of anesthetics, maintain hemodynamic stability, no respiratory inhibition, postoperative amnesia, anti-vomiting and diuretic effects [39]. Dexmedetomidine has been widely used and recognized in ICU sedation and general anesthesia because of its fast absorption, fast effect, complete metabolism [40, 41]. As a highly selective central α2 adrenergic receptor agonist, dexmedetomidine was sedative and analgesic effects and can produce synergistic effects with anesthetics and analgesics.

Dexmetomidine is a new choice of anesthetic auxiliary drugs, which provides effective sedation for ophthalmic surgery anesthesia patients, and can provide a certain degree of analgesic effect. At the same time, it has the effect of compliance amnesia, especially in sober sedation, it has no respiratory inhibition and less side effect which makes it show its unique superiority and application value in clinical anesthesia practice. Existing studies have confirmed that the sedative, hypnotic and anti-anxiety effects of dexmetomidine are dose-dependent [42]. Therefore, the application of dexmetomidine in ophthalmic anesthesia surgery has the following advantages: 1) to provide patients with satisfactory and comfortable sedation without reducing the degree of cooperation of intraoperative patients; 2) it can increase the tolerance of ophthalmic anesthesia patients to pain; 3) can maintain the stability of hemodynamics and reduce the degree of stress in intraoperative patients; 4) can eliminate the bad memory of ophthalmic local anesthesia patients with anesthesia and surgical operation, etc. It has a certain anterograde amnesia effect; 5) no increase in the incidence of postoperative adverse reactions.

Fentanyl is a classical opioid anesthetic with strong fat solubility, which can maintain the drug effect near 30 min after a single administration, and the blood concentration can show the second peak at 20~90 min [43]. Fentanyl can maintain analgesic effect for a long time after operation. These characteristics meet the clinical anesthesia needs of short operation time, no savings and rapid recovery after ophthalmic surgery in children. Studies have shown that the combination of fentanyl and sevoflurane in the anesthesia induction stage can produce synergistic effect and effectively improve the analgesic and sedative effect [44]. Therefore, the application of fentanyl in the induction stage of anesthesia combined with sevoflurane to maintain intraoperative anesthesia can give full play to the advantages of the two drugs, not only to maintain a good anesthetic effect, but also to recover quickly after operation. The drug has less savings in the body, and the use of fentanyl to induce a single low dose of the drug will not cause respiratory inhibition, and can maintain a longer postoperative analgesic effect, in order to alleviate the pain caused by the regression of sevoflurane during the recovery period [45]. It can effectively reduce the restlessness, crying and other adverse reactions in children. In this sudy, we found fentanyl combine with sevoflurane also can effectively reduce risk of emergence agitation in children undergoing ophthalmic surgery [46].

There are some limitations in this study. First of all, different doses included in the literature, different administration schemes, and different age of the patients resulted in clinical heterogeneity. Secondly, we only evaluated the incidence of EA, while the incidence of postoperative nausea and vomiting and other adverse reactions (such as dizziness, chills) could not be analyzed due to the lack of relevant data. Finally, the quality and quantity of the literature included are on the low side, which leads to the decrease of the test efficiency of the results of this study, the small sample size of the interventions included in the study, and the possible shortage of statistical efficiency may be insufficient. Based on the shortcomings of the existing research, clinicians should consider the influence of the above factors and choose carefully when applying the conclusions of this study.

## Conclusion

In summary, based on this study, the results of network meta analysis showed that dexmedetomidine, ketamine, propofol, fentanyl, midazolam, sufentanil, remifentanil or clonidine combine with sevoflurane also can effectively reduce risk of emergence agitation in children undergoing ophthalmic surgery compare wirh placebo. The effects of dexmedetomidine combine with sevoflurane in reducing risk of emergence agitation in children undergoing ophthalmic surgery was superior to other drugs.

## Data Availability

The analysed data sets generated during the study are available from the corresponding author on reasonable request.

## Abbreviations

EA: Emergence agitation
RCT: Randomized controlled trials
SUCRA: Surface under the cumulative ranking
